# Evidence of fibrinogen as a target of citrullination in IgM rheumatoid factor-positive polyarticular juvenile idiopathic arthritis

**DOI:** 10.1186/1546-0096-9-8

**Published:** 2011-03-25

**Authors:** Brooke E Gilliam, Melinda R Reed, Anil K Chauhan, Amanda B Dehlendorf, Terry L Moore

**Affiliations:** 1 Division of Adult and Pediatric Rheumatology, Saint Louis University School of Medicine, Saint Louis, Missouri USA; 2 Division of Adult and Pediatric Rheumatology, Saint Louis University School of Medicine, Room 211A Doisy Hall, 1402 South Grand Blvd, Saint Louis, Missouri 63104, USA

## Abstract

**Background:**

Several studies have noted the significance of measuring anti-cyclic citrullinated peptide (CCP) antibodies in juvenile idiopathic arthritis (JIA) as an important indicator for destructive disease, as is the case in rheumatoid arthritis (RA). While the role of anti-CCP antibodies in RA and JIA has become better understood, the identity of the target proteins of this modification has remained elusive. In this study, we evaluated serum from patients with various subtypes of JIA to investigate the presence of anti-deiminated (citrullinated) fibrinogen and anti-citrullinated α-enolase antibodies, and their association with RF and anti-CCP antibody isotypes.

**Methods:**

Sera were obtained from 96 JIA patients, 19 systemic lupus erythematosus (SLE) patients, and 10 healthy children. All sera were measured for antibodies against citrullinated and native fibrinogen and α-enolase by an enzyme linked immunosorbent assay (ELISA). In addition, all sera were assayed for anti-CCP antibody isotypes and rheumatoid factor (RF) isotypes by ELISA. The relationship between anti-citrullinated fibrinogen and anti-α-enolase antibodies and disease activity and joint damage were also investigated. All results were correlated with clinical and laboratory parameters using Spearman's rho correlation coefficient. Multiple logistic regression analysis was utilized to identify which variables were associated with joint erosions and diagnosis of JIA.

**Results:**

Thirty-one JIA patients (32%) demonstrated reactivity to citrullinated fibrinogen and 9 (9%) to citrullinated α-enolase. Reactivity to citrullinated fibrinogen and α-enolase was predominantly found in IgM RF-positive polyarthritis patients. Fourteen JIA patients reacted with native α-enolase and a higher percentage of SLE patients reacted with citrullinated α-enolase when compared to JIA patients. Anti-citrullinated fibrinogen antibodies correlated with the presence of IgG anti-CCP antibodies and IgA and IgM RF. The presence of anti-citrullinated α-enolase antibodies correlated with IgA anti-CCP antibodies. IgG anti-CCP antibodies were significantly associated with joint damage and anti-citrullinated fibrinogen antibodies were strongly associated with JIA when compared to control groups. Anti-citrullinated fibrinogen antibodies demonstrated high sensitivity (81%) for IgM RF-positive polyarticular JIA. IgG anti-CCP antibodies had the highest specificity (95%) for JIA, with anti-citrullinated fibrinogen antibodies, IgA anti-CCP antibodies and IgA RF all following at 84%.

**Conclusions:**

JIA patient sera exhibited strong reactivity to anti-citrullinated fibrinogen antibodies and demonstrated high sensitivity and specificity for JIA, primarily in IgM RF-positive polyarthritis patients. Fibrinogen is one of several protein targets for citrullination in JIA.

## Background

Anti-cyclic citrullinated peptide (anti-CCP) antibodies have been established as an important diagnostic tool in rheumatoid arthritis (RA), especially being demonstrated in patients with a more aggressive disease course [1]. The role of anti-CCP antibodies in juvenile idiopathic arthritis (JIA) remains undefined [2]. It has been shown that anti-CCP antibodies are less prevalent in JIA populations, mainly restricted to polyarthritis patients or patients with joint damage [3-6]. Several studies have noted the significance of measuring anti-CCP antibodies in JIA as an important indicator for destructive disease, as is the case in RA [3-9]. Recently, the identity of various immunoglobulin isotypes of anti-CCP antibodies has also been reported in RA [10] and JIA [11]. These studies revealed that a varied pattern of isotype usage early in disease was possibly associated with aggressive disease, especially when all three isotypes (IgG, IgA, and IgM) were present [2,10,11].

While the role of anti-CCP antibodies in RA and JIA has become better understood, the identity of the target proteins of this modification has remained elusive. Snir et al. [12] evaluated antibody reactivities to citrullinated proteins in RA patients to determine their prevalence and significance. They found that RA patients demonstrated varying antibody reactivities to citrullinated fibrinogen, citrullinated α-enolase, and the C1 epitope of citrullinated type II collagen (citC1^III^). Other studies have examined the significance of citrullinated fibrinogen [13-19], citrullinated α-enolase [13,20,21], citC1^III ^[13,22-24], and citrullinated vimentin [13,25-30] in RA and experimental arthritis. To date, only two studies on citrullinated vimentin has documented the identity and evaluated the significance of citrullinated proteins in a JIA population [31,32].

In this study, we evaluated serum from patients with various subtypes of JIA to investigate the presence of anti-deiminated (citrullinated) fibrinogen and anti-citrullinated α-enolase antibodies, and their association with RF and anti-CCP antibody isotypes. The aim of this study was to determine the prevalence and significance of previously identified target proteins for citrullination and elucidate their role in the disease process of JIA. Findings from this study point to fibrinogen as a target of citrullination in IgM RF-positive polyarticular JIA patients.

## Materials and methods

### Serum Samples

Sera were obtained from the Saint Louis University Pediatric Rheumatology Division outpatient clinics at the Saint Louis University Medical Center and Cardinal Glennon Children's Medical Center following informed consent. Sera were stored at -80°C until assayed. A total of 96 individual JIA serum samples (77 female and 19 male) were collected consecutively, including 16 with IgM RF-positive polyarthritis, 36 with IgM RF-negative polyarthritis, 25 with oligoarthritis, 13 with systemic-onset arthritis, 3 with psoriatic arthritis, and 3 with enthesitis-related arthritis. The JIA patients had a mean age of 11.2 ± 5.9 years, and mean disease duration of 3.9 ± 4.4 years (Table 1). In the JIA population, 88 patients (91.7%) were Caucasian, 3 (3.1%) were African American, 2 (2.1%) were Hispanic, and 3 (3.1%) were classified as other races. The patient demographics depict an accurate representation of the JIA population at our Pediatric Rheumatology outpatient clinics. Sixty-seven of the JIA patients had active disease at the time of sample collection, compared to 29 who were in disease remission. Radiographic joint damage was noted in 20 of 96 JIA patients. All JIA patients in this study fulfilled the International League of Associations of Rheumatology criteria [33,34].

**Table 1:** Demographic and laboratory features of JIA patients (n = 96)

Sex, no. females/males	77/19
Age, mean ± SD years (range)	11.2 ± 5.9 (2-17)
Disease Duration, mean ± SD years (range)	3.9 ± 4.4 (onset-16)
Tender/Swollen joint count, mean ± SD (range)	7.3 ± 5.8 (0-24)
No. patients with joint damage (%)	20 (20.8)
CRP, mean ± SD mg/dL (% positive)	1.56 ± 1.96 (39.3)
ESR, mean ± SD mm/hr (% positive)	27.13 ± 29.32 (52.1)

CRP: C-reactive protein; ESR: erythrocyte sedimentation rate.

Cut-points for a positive value: CRP (≥0.8 mg/dl), ESR (≥20 mm/hr).

Sera from 19 patients with pediatric-onset systemic lupus erythematosus (SLE) (17 female and 2 male) were collected consecutively from the outpatient clinics, following informed consent. The mean age of the SLE patients was 15.7 ± 3.1 years and the mean disease duration was 2.7 ± 3.2 years. Fifteen SLE patient (78.9%) were Caucasian, 2 (10.5%) were African American, 1 (5.3%) was Hispanic, and 1 (5.6%) was Filipino. Sera were also collected consecutively from 10 healthy children (9 female and 1 male) at the well-child clinic at Cardinal Glennon Children's Medical Center following informed consent. The mean age for the healthy children was 14.0 ± 5.9 years. Six healthy children (60.0%) were Caucasian, 2 (20.0%) were African American, and 2 were Asian (20.0%). The study was approved by the Institutional Review Board of the Saint Louis University Medical Center.

### Laboratory and clinical evaluation

Erythrocyte sedimentation rate (ESR) was determined by the modified Westergren technique and considered elevated at ≥15 mm/hr. C-reactive protein (CRP) was determined by electroimmunoassay and a value of ≥0.8 mg/dl was considered elevated. Mean levels of ESR and CRP are shown in Table 1. Initial determination of IgM RF positivity was performed by nephelometry or latex agglutination, which is how patients were classified for seropositive JIA. The QUANTA Lite RF enzyme-linked immunosorbent assays (ELISA) (Inova Diagnostics, Inc., San Diego, CA) were used for the detection of IgA and IgM RF according to the manufacturer's instructions. The cut-off value for positive IgA or IgM RF was 6 U. A third generation anti-CCP antibody test, the QUANTA Lite CCP3 ELISA (Inova Diagnostics, Inc., San Diego, CA), was used for the detection of IgG anti-CCP antibodies according to the manufacturer's instructions. The cut-off value for a positive result was 20 U. IgA and IgM anti-CCP antibodies were measured by ELISA (QUANTA Lite CCP3 ELISA, Inova Diagnostics, San Diego, CA) as previously described [11]. Cut-off values for a positive result were calculated at optical density (OD) = 0.16 and OD = 0.43, respectively, determined as the mean plus two standard deviations (SD) of a healthy population from a previous study [11]. Clinical data regarding signs of active disease (including joint pain and swelling, limitations of range of motion, fever, rash, visceral involvement, and inflammatory markers) were collected from patient records of the Pediatric Rheumatology clinics. Radiological data was evaluated for signs of joint damage (defined as joint space narrowing and/or erosions) by musculoskeletal radiologists and reviewed by the Pediatric Rheumatologists. Both clinical data and radiological data were collected from the same time period as serum was collected.

### In vitro deimination of human fibrinogen

Deimination of fibrinogen was performed as previously described [15,16]. Briefly, plasminogen-depleted human fibrinogen (95% pure, Calbiochem, Meudon, France) was purified by affinity chromatography on a protein-G column (HiTrap protein G, GE Healthcare, Piscataway, New Jersey) according to the manufacturer's instructions. Deimination was then performed using rabbit skeletal muscle peptidyl arginine deiminase (PAD) (Sigma, St. Louis, Missouri, USA, 7 U/mg fibrinogen) in 0.1 M Tris-HCl (pH 7.4), 10 mM CaCl_2_, 5 mM dithiothreitol for 2 hours at 37°C.

### Citrullinated and native fibrinogen ELISAs

ELISAs with citrullinated and native forms of fibrinogen were performed as previously described [15,16] with some modifications. Briefly, ninety-six-well microtitre plates (Nunc, Roskilde, Denmark) were coated with human native fibrinogen or human citrullinated fibrinogen (5 μg/ml) diluted in phosphate buffered saline (PBS) and incubated overnight at 4°C. The plates were then blocked with PBS containing 2% bovine serum albumin (BSA) (Sigma, St. Louis, Missouri, USA) for one hour at 4°C, followed by 3 washes with PBS/0.05% Tween. Patient sera, diluted 1:50 in 2 M NaCl PBS, were added to the wells in duplicate and incubated for one hour at room temperature (RT) with gentle agitation. After 3 washes with PBS/0.05% Tween, horseradish peroxidase (HRP)-labeled goat anti-human IgG (γ-chain specific) (Antibodies Incorporated, Davis, California, USA) diluted 1:15,000 was added to the wells and incubated for one hour at RT. After 3 washes with PBS/0.05% Tween, the amount of antibodies bound were detected with tetramethylbenzidine (TMB), an HRP substrate (BioFX, Owing Mills, Maryland, USA), and 0.25 M H_2_SO_4 _was used to stop the reaction. The OD was read at 450 nm (Tecan Group Ltd., Männedorf, Switzerland). Patient results from the duplicate wells were averaged and the OD from a blank well containing PBS/0.05% Tween was subtracted from the average. Serum was considered positive if the titer reached 2 SD above the mean for healthy controls. Positive cut-off points were OD = 0.59 for anti-citrullinated fibrinogen antibodies and OD = 0.46 for native fibrinogen.

### Citrullinated and native α-enolase ELISAs

Citrullinated and native α-enolase ELISAs were performed as previously described by Lundberg et al. [21] with some modifications. Briefly, 96-well microtitre plates (Nunc, Roskilde, Denmark) were coated with either a citrullinated α-enolase peptide (CKIHA-X-EIFDS-X-GNPTVEC, where X represents citrulline) or the arginine-containing control peptide (CKIHAREIFDSRGNPTVEC) (Bachem Americas Inc., Torrance, California, USA) at 5 μg/ml diluted in 50 mM carbonate buffer (pH 9.6) and incubated overnight at 4°C. Following 3 washes with PBS/0.1% Tween, the plates were blocked with 2% BSA diluted in PBS for one hour at RT. The plates were washed as described above. Serum samples were diluted 1:50 in radioimmunoassay (RIA) buffer (10 mM Tris, 1% BSA, 350 mM NaCl, 1% Triton-X-100, 0.5% sodium deoxycholate, 0.1% sodium dodecyl sulfate (SDS)), added in duplicate, and incubated for 90 minutes at RT with gentle agitation. After 3 washes with PBS/0.1% Tween, the plates were incubated for one hour at RT with gentle agitation with HRP-conjugated goat anti-human IgG (γ-chain specific) (Antibodies Incorporated, Davis, California, USA), diluted 1:10,000 in RIA buffer. After a final wash step, bound antibodies were detected with TMB and the reaction was stopped by the addition of 0.25 M H_2_SO_4_. The absorbance was measured at 450 nm using a Tecan plate reader (Tecan Group Ltd., Männedorf, Switzerland). Patient results from the duplicate wells were averaged and the OD from a blank well containing PBS/0.05% Tween was subtracted from the average. Serum was considered positive if the titer reached 2 SD above the mean for healthy controls. Cut-off values were considered positive at OD = 0.91 for anti-citrullinated α-enolase antibodies and OD = 0.55 for native α-enolase.

### Statistical Analyses

Patient groups were compared using Student's *t *test and χ^2 ^test for proportions. For tables with cells with small frequencies, Fisher's exact test was used. Comparisons between JIA subtypes and control groups were performed with one-way ANOVA followed by Bonferroni *post hoc *analysis. Correlations were analyzed using Spearman's rho correlation coefficient. Multiple logistic regression analysis was utilized to identify which variables were associated with joint erosions and diagnosis of JIA. Variables associated with joint damage and JIA in univariate analysis were included in regression analysis. Results were expressed as odds ratios (OR) with 95% confidence intervals (CI) and p-values. The sensitivity, specificity, and positive predictive value (PPV) of citrullinated fibrinogen, citrullinated α-enolase, anti-CCP antibody isotypes, and RF isotypes were calculated in the JIA population. The sensitivity expresses the percentage of JIA patients positive for the test and specificity expresses the frequency of negative tests in the absence of JIA or JIA subtypes. PPV describes the group of patients with a positive test result who are correctly diagnosed. Statistical analyses were carried out using SPSS version 15.0 (Chicago, IL, USA). A p-value < 0.05 was considered statistically significant.

## Results

### JIA serum reactivity to native and citrullinated fibrinogen and α-enolase

**Figure 1:**
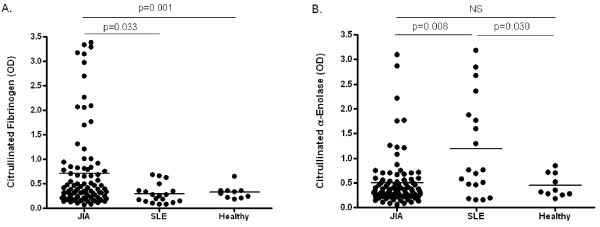
**Levels of anti-citrullinated fibrinogen and g-enolase antibodies in JIA, SLE, and healthy children**. IgG antibody recognition of citrullinated fibrinogen (a) and citrullinated α-enolase (b) by ELISA in sera from JIA patients (n = 96), SLE patients (n = 19), and healthy children (n = 10). Bars indicate the median levels of citrullinated antibodies in each group. P-values were considered statistically significant at < 0.05. OD: optical density.

Thirty-one JIA patients (32.3%) showed reactivity to citrullinated fibrinogen (Table 2). IgM RF-positive polyarthritis patients demonstrated the highest level of reactivity with citrullinated fibrinogen (Table 2), which was significantly higher than other JIA subtypes and healthy and SLE control groups (Table 3). Seven of the 11 (63.6%) IgM RF-negative polyarthritis patients who were positive for anti-citrullinated fibrinogen antibodies were also positive for IgM RF by ELISA. Anti-citrullinated fibrinogen antibodies were significantly elevated in JIA patients when compared to SLE and healthy children (Figure 1A). Seven (7.3%) JIA patients reacted with native fibrinogen, including one with IgM RF-positive polyarthritis, 3 with IgM RF-negative polyarthritis, and 3 with oligoarthritis.

**Table 2:** Antibody concentration and positivity in JIA subtypes (n = 96)

	Poly RF+ n = 16 Mean ± SD	Poly RF- n = 36 Mean ± SD	Oligo n = 25 Mean ± SD	Systemic n = 13 Mean ± SD	Enthesitis n = 3 Mean	Psoriatic n = 3 Mean
**Citrullinated Fibrinogen (OD)** Positivity n(%)	1.6 ± 1.1 13 (81.3%)	0.60 ± 0.75 11(30.6%)	0.54 ± 0.65 6(24.0%)	0.36 ± 0.18 1(7.7%)	0.37 0(0%)	0.28 0(0%)
**Citrullinated α-Enolase (OD)** Positivity n(%)	0.54 ± 0.49 3(18.8%)	0.47 ± 0.51 2(5.6%)	0.35 ± 0.16 0(0%)	0.40 ± 0.24 1(7.7%)	1.6 2(66.7%)	1.2 1(33.3%)
**IgG CCP (U)** Positivity n(%)	109.9 ± 116.4 9(56.3%)	18.3 ± 63.96 5(13.9%)	0.76 ± 12.7 0(0%)	4.5 ± 11.4 0(0%)	3.7 0(0%)	1.6 0(0%)
**IgA CCP (OD)** Positivity n(%)	0.28 ± 0.56 4(25.0%)	0.11 ± 0.21 6(16.7%)	0.04 ± 0.02 0(0%)	0.04 ± 0.04 0(0%)	0.09 0(0%)	0.04 0(0%)
**IgM CCP (OD)** Positivity n(%)	0.91 ± 1.2 5(31.3%)	0.53 ± 0.77 10(27.8%)	0.17 ± 0.09 0(0%)	0.20 ± 0.12 1(7.7%)	0.13 0(0%)	0.31 0(0%)
**IgA RF (U)** Positivity n(%)	19.4 ± 29.8 10(62.5%)	1.7 ± 4.5 4(11.1%)	1.1 ± 2.4 0(0%)	0.99 ± 3.4 1(7.7%)	2.1 0(0%)	0.15 0(0%)
**IgM RF (U)** Positivity n(%)	66.3 ± 52.4 16(100%)	14.8 ± 23.5 17(47.2%)	3.6 ± 11.0 5(20.0%)	15.2 ± 18.1 8(61.5%)	3.3 0(0%)	2.9 0(0%)

Continuous variables are expressed as means ± SD. Anti-CCP antibodies: anti-cyclic citrullinated peptide antibodies; CRP: C-reactive protein; ESR: erythrocyte sedimentation rate; n: number of patients; OD: optical density; RF: rheumatoid factor; U: Units. Cut points for positive values: Anti-citrullinated fibrinogen antibodies (OD≥0.59), Anti-citrullinated α-enolase antibodies (OD≥0.91), IgG anti-CCP antibodies (≥20U), IgA anti-CCP antibodies (OD≥0.16), IgM anti-CCP antibodies (OD≥0.43), IgA RF (≥6U), IgM RF (≥6U).

**Table 3:** Significant differences in anti-citrullinated fibrinogen antibody levels in JIA subtypes and healthy and SLE control groups

Study Group	JIA subtypes and Control Groups	Citrullinated-Fibrinogen p-value
IgM RF-positive polyarthritis	IgM RF-negative polyarthritis	0.002
	Oligoarthritis	0.001
	Systemic-onset arthritis	< 0.001
	Psoriatic arthritis	0.049
	Enthesitis-related arthritis	0.049
	Healthy	0.001
	SLE	< 0.001

Bonferroni *post hoc *analysis of association between anti-citrullinated fibrinogen levels in JIA subtypes and control groups. Significant differences at p < 0.05. JIA: juvenile idiopathic arthritis; RF: rheumatoid factor; SLE: systemic lupus erythematosus.

Nine (9.4%) JIA patients showed reactivity to citrullinated α-enolase (Table 2). SLE patients exhibited significantly higher reactivity with citrullinated α-enolase in comparison to JIA and healthy children (Figure 1B). Fourteen (14.6%) JIA patients reacted with native α-enolase, including 1 with IgM RF-positive polyarthritis, 9 with IgM RF-negative polyarthritis, 2 with oligoarthritis, and 2 with enthesitis-related arthritis.

Five JIA patients reacted with both citrullinated fibrinogen and citrullinated α-enolase, including 3 with IgM RF-positive polyarthritis, one with IgM RF-negative polyarthritis, and one with systemic-onset JIA. All 3 IgM RF-positive JIA patients had active disease, with elevated ESR. Additionally, 2/3 IgM RF-positive polyarthritis patients were positive for IgA RF and anti-CCP antibody isotypes. The patient with systemic-onset JIA had active disease, high ESR, and macrophage activation syndrome in the past and now manifested purely polyarticular disease. The IgM RF-negative polyarthritis patient was asymptomatic at the time of sample collection, but still demonstrated elevated levels of IgA and IgG anti-CCP antibodies.

### Commonality between anti-citrullinated antibody reactivities in JIA

**Figure 2:**
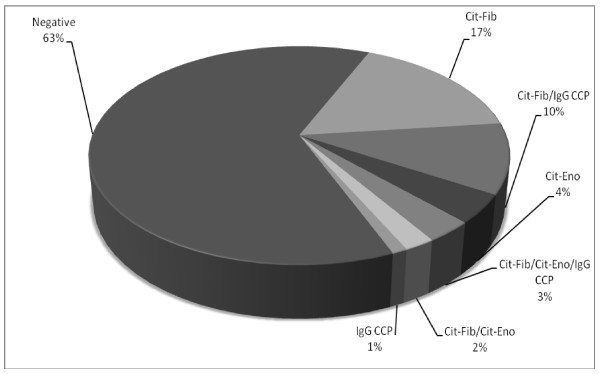
**Patterns of citrullinated antibody reactivity in JIA sera (n = 96)**. Cit-Eno: anti-citrullinated α-enolase antibodies; Cit-fib: anti-citrullinated fibrinogen antibodies; IgG CCP: IgG anti-cyclic citrullinated peptide antibodies.

Antibodies against various citrullinated proteins may be present in JIA sera at the same time. Figure 2 illustrates the antibody reactivity pattern in this JIA cohort. The prominent subgroup in JIA included patients who reacted only with citrullinated fibrinogen (17%). Ninety-three percent of JIA patients positive for IgG anti-CCP antibodies also reacted with citrullinated fibrinogen, making up 10% of the JIA population (Figure 2). Three (3%) IgM RF-positive polyarthritis patients were positive for IgG anti-CCP antibodies and both anti-citrullinated fibrinogen and α-enolase antibodies (Figure 2).

### Clinical and serological correlations

Anti-citrullinated fibrinogen antibodies correlated significantly with anti-citrullinated α-enolase antibody levels (r = 0.35, p = 0.001), IgG anti-CCP antibodies (r = 0.55, p = 0.001), and IgA and IgM RF (r = 0.40 and r = 0.52, respectively; p = 0.001). No significant correlations were noted with anti-citrullinated fibrinogen antibodies and disease duration, patient age, joint damage, disease activity, ESR, or CRP. Anti-citrullinated α-enolase antibodies levels correlated significantly with IgA anti-CCP antibodies (r = 0.31, p = 0.007) in JIA. Additionally, a correlation between patient age and anti-citrullinated α-enolase antibodies were also noted (r = 0.30, p = 0.001). No significant correlations were found between anti-citrullinated α-enolase antibodies and disease duration, joint damage, disease activity, ESR, or CRP. Similar evaluation with native fibrinogen and native α-enolase yielded no statistically significant results.

Levels of IgG, IgM, and IgA anti-CCP antibodies, and IgA and IgM RF were all significantly elevated in patients with a positive result for anti-citrullinated fibrinogen antibodies versus patients who were negative for anti-citrullinated fibrinogen antibodies (Table 4).

**Table 4:** Significant differences in antibody levels in JIA patients positive versus negative for anti-citrullinated fibrinogen antibodies

	Positive for Anti-Citrullinated Fibrinogen Antibodies n = 31	Negative for Anti-Citrullinated Fibrinogen Antibodies n = 65	P-Value
**IgM RF (U)**	42.86	6.59	< 0.001
**IgA RF (U)**	15.27	1.25	0.005
**IgG anti-CCP Antibodies (U)**	65.64	0.86	0.001
**IgA anti-CCP Antibodies (OD)**	0.22	0.05	0.026
**IgM anti-CCP Antibodies (OD)**	0.69	0.32	0.009

Anti-CCP antibodies: anti-cyclic citrullinated peptide antibodies; n = number of patients; OD = optical density; RF = rheumatoid factor; U: Units. Cut points for positive values: Anti-citrullinated fibrinogen antibodies (OD≥0.59), IgG anti-CCP antibodies (≥20U), IgA anti-CCP antibodies (OD≥0.16), IgM anti-CCP antibodies (OD≥0.43), IgA RF (≥6U), IgM RF (≥6U). P-value < 0.05 was considered statistically significant.

### Relationship between serological markers and disease course

Mean levels of IgG anti-CCP antibodies and IgM RF were significantly elevated in JIA patients with joint damage (60.6U and 37.0U, respectively) compared to those with no joint damage (15.2U and 14.7U, respectively) (p < 0.05). IgA anti-CCP antibodies, IgA RF, and anti-citrullinated fibrinogen antibodies were also elevated in patients with joint damage, but did not reach statistical significance. IgG anti-CCP antibodies, IgA anti-CCP antibodies, IgA RF, IgM RF, and anti-citrullinated fibrinogen antibodies were included in logistic regression analysis. Regression analysis showed that IgG anti-CCP antibodies (OR = 5.3 [95% CI 1.6-17.7] p = 0.007) were the independent factor associated with joint damage in JIA.

When evaluating disease activity, mean levels of CRP and ESR were significantly elevated in patients with active disease (p < 0.01) and both were included in regression analysis. ESR (OR = 3.2 [95% CI 1.4-7.3] p = 0.006) was the independent factor associated with active disease in this JIA cohort. No significant differences were observed between anti-citrullinated fibrinogen antibodies and anti-citrullinated α-enolase antibodies when assessing disease activity in JIA.

Significantly elevated mean levels IgG anti-CCP antibodies and anti-citrullinated fibrinogen antibodies were observed in JIA patients compared to the control patient groups (SLE and healthy combined) (p < 0.05). As previously indicated, mean anti-citrullinated α-enolase antibody levels were significantly elevated in the control group, mainly SLE patients, compared to the JIA population (p < 0.05). When including IgG anti-CCP antibodies, anti-citrullinated fibrinogen antibodies, and anti-citrullinated α-enolase antibodies in multiple logistic regression analysis, anti-citrullinated fibrinogen antibodies were significantly correlated with JIA (OR = 14.8 [95% CI 1.4-160.9] p = 0.027), while anti-citrullinated α-enolase antibodies demonstrated an inverse relationship with JIA (OR = 0.29 [95% CI 0.13-0.64] p = 0.002).

### Sensitivity and specificity of anti-citrullinated fibrinogen and α-enolase antibodies for JIA

The sensitivity, specificity, and PPV of anti-citrullinated fibrinogen antibodies, anti-citrullinated α-enolase antibodies, IgG and IgA anti-CCP antibodies, and IgA and IgM RF for JIA subtypes are shown in Table 5. Sensitivities, specificities and PPVs were calculated for the overall JIA population and for each subtype, excluding enthesitis-related JIA and psoriatic JIA due to small sample size. Overall sensitivities ranged from 9.4% to 47.9% and specificities from 57.9% to 94.7%. IgM RF and anti-citrullinated fibrinogen antibodies demonstrated the strongest sensitivity for JIA overall (47.9% and 32.3%, respectively). Anti-citrullinated fibrinogen antibodies demonstrated high sensitivity for IgM RF-positive polyarthritis at 81.3%. IgG and IgA anti-CCP antibodies (94.7% and 84.2%, respectively), anti-citrullinated fibrinogen antibodies (84.2%), and IgA RF (84.2%) exhibited the highest specificity for JIA. IgG anti-CCP antibodies and anti-citrullinated fibrinogen antibodies demonstrated the highest PPV for JIA overall and IgG anti-CCP antibodies and IgA RF in IgM RF-positive polyarticular JIA (Table 5).

**Table 5:** Sensitivity, Specificity, and PPV of anti-citrullinated antibodies and RF isotypes for the overall JIA population and JIA subtypes

	Sensitivity (%) With 95%CI	Specificity (%) With 95% CI	PPV (%) With 95% CI
**Anti-Citrullinated Fibrinogen Antibodies (> 0.59OD)**			
**Overall**	32.3 (23.3-42.7)	84.2 (59.5-95.8)	91.2 (75.2-97.7)
**Poly RF-positive**	81.3 (53.7-95.0)	78.8 (69.2-86.1)	38.2 (22.7-56.4)
**Poly RF-negative**	30.6 (16.9-48.3)	70.9 (59.4-80.3)	32.4 (18.0-50.6)
**Oligoarthritis**	24.0 (10.2-45.5)	68.9 (58.1-78.0)	17.6 (7.4-35.0)
**Systemic-Onset**	7.7 (0.4-37.9)	67.6 (57.6-76.4)	29.4 (0.1-17.1)
**Anti-Citrullinated α-Enolase Antibodies (> 0.91OD)**			
**Overall**	9.4 (4.6-17.5)	57.9 (34.0-78.9)	52.9 (28.5-76.1)
**Poly RF-positive**	18.8 (5.0-46.3)	85.9 (77.1-91.8)	17.6 (4.6-44.2)
**Poly RF-negative**	5.6 (1.0-20.0)	70.9 (59.4-80.3)	11.8 (2.1-37.7)
**Oligoarthritis**	0	81.1 (71.2-88.3)	0
**Systemic-Onset**	7.7 (0.4-37.9)	84.3 (75.5-90.5)	5.9 (0.3-31.0)
**IgG anti-CCP antibodies (> 20U)**			
**Overall**	14.6 (8.5-23.6)	94.7 (71.9-99.7)	93.3 (66.0-99.7)
**Poly RF-positive**	56.3 (30.6-79.2)	93.9 (86.8-97.5)	60.0 (32.9-82.5)
**Poly RF-negative**	13.9 (5.2-30.3)	87.3 (77.5-93.4)	33.3 (13.0-61.3)
**Oligoarthritis**	0	83.3 (73.7-90.0)	0
**Systemic-Onset**	0	85.3 (76.6-91.2)	0
**IgA anti-CCP antibodies (> 0.16OD)**			
**Overall**	10.4 (5.4-18.7)	84.2 (59.5-95.8)	76.9 (46.0-93.8)
**Poly RF-positive**	25.0 (8.3-52.6)	90.9 (83.0-95.5)	30.8 (10.3-61.1)
**Poly RF-negative**	16.7 (6.9-33.5)	91.1 (82.0-96.1)	46.2 (20.4-73.9)
**Oligoarthritis**	0	85.6 (76.2-91.8)	0
**Systemic-Onset**	0	87.3 (78.8-92.8)	0
**IgM Anti-CCP Antibodies (> 0.45OD)**			
**Overall**	16.7 (10.1-26.0)	73.7 (48.6-89.9)	76.2 (52.5-90.9)
**Poly RF-positive**	31.3 (12.1-58.5)	83.8 (74.8-90.2)	23.8 (9.1-47.5)
**Poly RF-negative**	16.7 (6.9-33.5)	91.1 (82.0-96.1)	46.2 (20.4-73.9)
**Oligoarthritis**	0	76.7 (66.3-84.7)	0
**Systemic-Onset**	7.7 (0.4-37.9)	80.4 (71.1-87.3)	4.8 (0.2-25.9)
**IgA RF (> 6U)**			
**Overall**	15.6 (9.3-24.8)	84.2 (59.5-95.8)	83.3 (57.7-95.6)
**Poly RF-positive**	62.5 (35.9-83.7)	91.9 (84.2-96.2)	55.6 (31.3-77.6)
**Poly RF-negative**	11.1 (3.6-27.0)	82.2 (71.7-89.6)	22.2 (7.4-48.1)
**Oligoarthritis**	0	80.0 (70.0-87.4)	0
**Systemic-Onset**	7.7 (0.4-37.9)	83.3 (74.4-89.7)	5.6 (0.3-29.4)
**IgM RF (> 6U)**			
**Overall**	47.9 (37.7-58.3)	63.1 (38.6-82.8)	86.8 (74.0-94.1)
**Poly RF-positive**	100.0 (75.9-100.0)	62.6 (52.3-72.0)	30.2 (18.7-44.5)
**Poly RF-negative**	47.2 (30.8-64.7)	54.4 (42.9-65.5)	32.1 (20.3-46.4)
**Oligoarthritis**	20.0 (7.6-41.3)	46.7 (36.2-57.4)	9.4 (3.5-21.4)
**Systemic-Onset**	61.5 (32.3-84.9)	55.9 (45.7-65.6)	15.1 (7.2-28.1)

Anti-CCP antibodies: anti-cyclic citrullinated peptide antibodies; OD: optical density; CI: confidence interval; PPV: positive predictive value; RF: rheumatoid factor; U: units. Subtypes with small sample size were not included in the subtype analysis.

## Discussion

Several studies in RA and JIA have evaluated the potential role of anti-CCP antibodies in disease pathogenesis [1,2]. Recent studies in RA have attempted to identify the target proteins for citrullination [12]. In RA, fibrinogen has been identified as one of the protein targets for this modification and a potential marker of disease and predictor of radiographic progression [14,17]. We observed elevated levels of anti-citrullinated fibrinogen antibodies in 31/96 (32%) of the JIA population, most commonly in IgM RF-positive polyarthritis patients (81%). The prevalence of anti-citrullinated fibrinogen antibodies within the IgM RF-positive polyarthritis subtype was similar to findings in RA, with ranges from 48% to 82% [12,18,19]. Moreover, the significant relationship between IgG anti-CCP antibodies and IgM RF with anti-citrullinated fibrinogen antibodies has been shown in adult RA [12-14], similar to what we have documented in our cohort of IgM RF-positive polyarticular JIA patients. Varying serological patterns of reactivity were noted in JIA, with the most frequent combination being IgG anti-CCP antibodies and anti-citrullinated fibrinogen antibodies, or anti-citrullinated fibrinogen antibodies alone. Similar findings have been reported in RA [12]. This again adds credence that the IgM RF-positive polyarticular JIA onset-type patients are similar to adult RA and the potential for life long disease.

Previous studies have shown association between α-enolase and various autoimmune diseases, including SLE, systemic sclerosis, Behçet's disease, mixed cryoglobulinemia, autoimmune hepatitis, and primary sclerosing cholangitis [35-37]. Anti-α-enolase antibodies have also been associated with renal involvement in SLE [35]. This documentation of α-enolase in various autoimmune diseases may also explain the high prevalence of native α-enolase in the JIA population. Further evaluation is required to determine the significance of citrullinated and native α-enolase in the SLE and JIA populations.

There were some limitations to our current study. The size of the healthy population was small and the control groups and JIA patients were not precisely matched by age or gender. Though the group of healthy children was small, the cut-off values generated from this group provided a range of results similar to previous studies in RA [12,18,19]. While the present results indicate measurement of anti-citrullinated fibrinogen antibodies as a potential marker for identifying patients with more aggressive disease, such as those with IgM RF-positive polyarthritis, longitudinal studies with early-onset JIA patients are necessary to further verify this claim.

Measurement of all 3 anti-CCP antibody isotypes has been suggested in both RA [10] and JIA [11]. Verpoort et al. [10] found that undifferentiated arthritis patients who were positive for both IgA and IgM anti-CCP antibodies were more likely to develop RA than those with IgG anti-CCP antibodies alone. We previously noted that the presence of all 3 anti-CCP antibody isotypes in JIA patients correlated with more severe disease [11]. The present study demonstrated strong specificities for IgG and IgA anti-CCP antibodies in the overall JIA population, in addition to high specificities in the polyarticular JIA patients. This data further supports measuring anti-CCP antibody isotypes in JIA patients, particular those patients with polyarticular disease. Measuring IgM RF by ELISA has been shown to be a more sensitive method of measurement than latex agglutination or nephelometry [2]. In agreement with several studies in JIA [2], increased sensitivity was observed when measuring IgM RF by ELISA, explaining an increased prevalence of IgM RF in patients diagnosed with IgM RF-negative polyarthritis, systemic-onset JIA, and oligoarthritis. In addition, our JIA patients initially diagnosed with systemic-onset JIA later developed diffuse polyarticular disease and then IgM RF positivity on several evaluations, originally being IgM RF negative by nephelometry or latex agglutination.

IgG anti-CCP antibodies and IgM RF had the strongest association with radiographic progression in JIA. Fifty percent of JIA patients with joint damage demonstrated increased reactivity to anti-citrullinated fibrinogen antibodies; however, no statistical correlation was noted. A conclusion can be drawn that while anti-citrullinated fibrinogen antibodies were prevalent in JIA, other proteins may be citrullinated, and could possibly demonstrate stronger association with joint damage. Further evaluation of various target proteins, including vimentin and type II collagen, are necessary to determine the significance of this modification in JIA pathogenesis. These studies are currently underway in our laboratory.

## Conclusions

Of the antibodies measured in this study, anti-citrullinated fibrinogen antibodies showed the strongest association with JIA when compared to healthy and SLE control groups. Additionally, anti-citrullinated fibrinogen antibodies demonstrated high sensitivity for IgM RF-positive polyarthritis patients, along with anti-CCP antibody isotypes and IgM RF demonstrating high specificities. Our data would suggest that measuring anti-citrullinated fibrinogen antibodies, in addition to anti-CCP antibody isotypes and IgM RF, may be beneficial in identifying patients that will develop more aggressive disease. For the first time, we have identified fibrinogen as a potential target for citrullination in JIA, particularly in patients with IgM RF-positive polyarticular JIA.

## List Of Abbreviations

BSA: bovine serum albumin; CCP: cyclic citrullinated peptide; CI: confidence interval; CRP C: - reactive protein; ELISA enzyme-linked immunosorbent assay; ESR: erythrocyte sedimentation rate; HRP: horseradish peroxidase; JIA: juvenile idiopathic arthritis; OD: optical density; OR: odds ratio; PAD: peptidyl arginine deiminase; PPV: positive predictive value; RA: rheumatoid arthritis; RF: rheumatoid factor; RIA: radioimmunoassay; RT: room temperature; SDS: sodium dodecyl sulfate; SLE: systemic lupus erythematosus; SPSS: statistical package social sciences; TMB: tetramethylbenzidene.

## Competing interests

The authors declare that they have no competing interests.

## Authors' contributions

BEG conceived the study, participated in the design of the study, performed immunoassays, performed data analysis, and drafted the manuscript. MRR participated in the design of the study, performed immunoassays, performed data analysis, acquired patient information, and drafted the manuscript. AKC participated in the design of the study and revised the manuscript. ABD performed immunoassays and acquired patient information. TLM conceived the study, participated in the design of the study, and drafted the manuscript. All authors have read and approved the final manuscript.

## Author Details

**BEG**, M.S, is a research assistant at the Division of Adult and Pediatric Rheumatology, Saint Louis University School of Medicine. **MRR**, M.D, is the Assistant Clinical Professor of Internal Medicine at the Division of Adult and Pediatric Rheumatology, Saint Louis University School of Medicine. **AKC**, PhD, is a Visiting Professor at the Division of Adult and Pediatric Rheumatology, Saint Louis University School of Medicine. **ABD**, M.D, is a Subspecialty Resident at the Division of Adult and Pediatric Rheumatology, Saint Louis University School of Medicine. **TLM**, M.D, is the Director, Division of Adult and Pediatric Rheumatology and a Professor of Internal Medicine, Pediatrics, and Molecular Microbiology and Immunology at the Saint Louis University School of Medicine.
